# Adequacy of Pathologic Reports of Invasive Breast Cancer From Mastectomy Specimens at Tikur Anbessa Specialized Hospital Oncology Center in Ethiopia

**DOI:** 10.1200/JGO.17.00198

**Published:** 2018-08-01

**Authors:** Abdu A. Yesufe, Mathewos Assefa, Abebe Bekele, Wondwossen Ergete, Abreha Aynalem, Tigeneh Wondemagegnehu, Johan Tausjø, Gizachew Assefa Tessema, Eva Johanna Kantelhardt, Ted Gansler, Ahmedin Jemal

**Affiliations:** **Abdu A. Yesufe**, **Mathewos Assefa**, **Abebe Bekele**, **Wondwossen Ergete**, **Abreha Aynalem**, and **Tigeneh Wondemagegnehu**, Addis Ababa University, Tikur Anbessa Specialized Hospital, Addis Ababa; **Gizachew Assefa Tessema**, University of Gondar, Gondar, Ethiopia; **Johan Tausjø**, Norwegian Radium Hospital, Oslo University, Oslo, Norway; **Eva Johanna Kantelhardt**, Institute of Medical Epidemiology, Martin-Luther-University, Halle an der Saale, Germany; and **Ted Gansler** and **Ahmedin Jemal**, American Cancer Society, Atlanta, GA.

## Abstract

**Purpose:**

Although information from pathology reports is essential to the care of individuals with cancer and to population-level cancer control, no systematic evidence exists regarding the adequacy of breast pathology reporting in Ethiopia. This study audited pathology reports of mastectomy specimens from patients evaluated at the Tikur Anbessa Specialized Hospital Oncology Center in Addis Ababa, Ethiopia.

**Methods:**

Mastectomy pathology reports from February 2014 through January 2016 were assessed for gross and microscopic information considered by the Breast Cancer Initiative 2.5 (BCI 2.5; formerly the Breast Health Global Initiative) guideline to be necessary for care of patients with breast cancer stratified according to basic, limited, and enhanced resource settings.

**Results:**

Fewer than two thirds (61.6%) of the 417 reports we reviewed included all four of the BCI 2.5 basic pathology data elements we could evaluate with available data (tumor category, lymph node category, histologic type, and histologic grade). Only 1.0% of reports included all three pathology data elements recommended for limited resource settings (estrogen receptor status, margin status, and lymphovascular invasion). Several elements were significantly more likely to be noted in reports from nonpublic hospitals than from public hospitals. Although only three of 417 reports included checklists or templates, all three of these reports included all of the basic pathology information, and they all included at least two of the three limited pathology elements not already on the basic list.

**Conclusion:**

More than one third (38.4%) of mastectomy pathology reports did not meet BCI 2.5 standards for basic resource settings. Quality measurement and improvement programs and capacity-building interventions by national pathology and oncology organizations, collaboration with medical and public health organizations in neighboring countries, adoption of synoptic reporting templates, use of electronic pathology reporting, and histotechnology and histopathology training collaborations with laboratories in high-resource regions are recommended.

## INTRODUCTION

Breast cancer is the most commonly diagnosed malignancy and the leading cause of cancer death in women worldwide and in most low- and middle-income countries (LMICs), including Ethiopia. As a result of reproduction and lifestyle changes, as well as the growth and aging of the population, the total number of patients is rapidly increasing in LMICs.^1-3^

Accurate and complete pathology reporting of breast surgical specimens is vital to determine whether lesions are benign or malignant, assure completeness of surgery, estimate the risk of cancer recurrence, and select appropriate treatment tailored to tumor characteristics.^4-6^ Oncologic pathology reporting is also essential to cancer registries and their role in guiding national and regional cancer control policies.^7,8^

Pathology and oncology organizations in several high-resource nations created recommendations for information to be included in pathology reports for nearly all types of cancer.^9-11^ However, consistently obtaining some of the information required for breast cancer pathology protocols is not feasible in low-resource settings. Furthermore, some of this information (eg, predictive markers for molecular targeted therapies) may not be relevant to treatments available in LMICs. For these reasons, international collaborations such as the Breast Health Global Initiative (BHGI) and its successor Breast Cancer Initiative 2.5 (BCI 2.5) developed recommendations for breast cancer pathology (and other components of breast cancer control) for settings with basic, limited, enhanced and maximal health care resources. The basic metrics relevant to completeness of pathology reports from mastectomy specimens are tumor histologic type, histologic grade, size of tumor (T) category, and nodal involvement (N) category. In addition to these basic recommendations, assessment of margin status and lymphovascular invasion, and immunohistochemical (IHC) determination of estrogen receptor (ER) status, are recommendations relevant to histopathology reports in settings with limited resources. Sentinel lymph node mapping and assessment of ductal carcinoma in situ extent accompanying an invasive cancer are also noted in the 2017 BCI 2.5 guideline, but these recommendations were not, to the best of our knowledge, in effect during the period between 2014 and 2016, from which patients included in this study were diagnosed. In addition to basic and limited data elements, enhanced recommendations include determinations of human epidermal growth factor receptor 2 (HER2) overexpression or gene amplification and IHC determination of progesterone receptor (PR) status. The recommendations for maximal resource settings are not relevant to this study.^12-15^

Although quite a few studies have evaluated the completeness of breast cancer pathology reports in high-resource settings over the past three decades,^5,16-20^ to the best of our knowledge, only two published reports described similar audits in sub-Saharan Africa.^21,22^ Both of these studies identified substantial gaps in documentation of BHGI basic pathology information.^12-14^

In high-resource settings, quality measurement and quality improvement programs are facilitated by availability of infrastructure, such as electronic medical records and laboratory information systems, and by nationwide organizations of clinicians involved in cancer care. For example, the American College of Surgeons Commission on Cancer standards currently mandate that 95% of pathology reports from approved facilities contain all the scientifically validated elements of the College of American Pathologists checklists.^23^ Another example is the Cancer Care Ontario program, which provides feedback to hospitals and pathologists on pathology report completeness, based on assessment of reports submitted electronically to the Ontario Cancer Registry; this program was successful in increasing the proportion of synoptic reports of breast, colorectal, prostate, lung, and endometrial cancer resection specimens to > 90%.^24^

Between 2005 and 2010, there had been an attempt to implement BHGI guidelines at the Tikur Anbessa Specialized Hospital Oncology Center (TASHOC) in Addis Ababa, Ethiopia, during a project that provided endocrine treatment (free for all patients), mammography, and procurement of some equipment.^25^ In the recent clinical experience of the authors of this study, breast cancer pathology reports in Ethiopia often lack data elements that are necessary for staging and treatment planning. However, no systematic evidence currently exists regarding the adequacy of breast pathology reporting in Ethiopia. This study was undertaken to evaluate the adequacy of pathology reports of mastectomy specimens from patients registered at TASHOC and thereby assess whether concerns based on the authors’ clinical observations are justified. By demonstrating gaps in pathology reporting quality and their implications for treatment decisions and health outcomes to professional and regulatory bodies in Ethiopia, the findings from this study will be a point of departure for developing and implementing a national standard of care in breast histopathology and for related quality improvement interventions.

## METHODS

### Study Design, Population, and Data Collection

This institution-based cross-sectional study was approved by the Addis Ababa University institutional review board. Records for all patients with invasive breast cancer evaluated at the TASHOC from February 2014 through January 2016 were reviewed. This study was limited to patients treated by mastectomy with available pathology reports of the mastectomy specimen from TASHOC and referring hospitals from all over the country. Patients with pathology reports only from core needle biopsy, fine-needle aspiration cytology, or incisional or excisional biopsy were excluded. We also excluded patients who had distant metastasis at presentation, because some pathologists might have assumed that certain findings were less relevant to clinical management of these patients. Pretest of the data extraction form was done by the principal investigator (A.A.Y.) on 40 pathology reports of mastectomy specimens, which were not included in the study, and the data extraction procedure and form were modified based on the pretest results. Two days of training were provided for the data collection and extraction team by the principal investigator.

### Variables

Pathology reports of eligible patients were assessed for the presence or absence of BCI 2.5 basic, limited, and enhanced pathology information (the maximal resource was not considered). The basic information addressed in our study consists of the pathologic T and N categories and Nottingham combined histologic grade according to the seventh edition American Joint Committee on Cancer and Union for International Cancer Control staging systems^26,27^ and the tumor histologic type. N categories could not be precisely assigned when < 10 lymph nodes from axillary lymph node dissections were examined; if any involved nodes were identified, these patients were classified as node positive (N+). Patients with 10 or more lymph nodes examined were classified as N0, N1, N2, or N3.

Additional information recorded from the pathology reports included patient age and sex, tumor location (laterality and quadrant), presence or absence of brief clinical history, whether the report included a template or synoptic summary (as opposed to only a narrative report), and whether the specimen was interpreted by a practicing pathologist only or by a pathology resident supervised by a senior pathologist. Hospitals generating the pathology reports were initially classified as public teaching, public nonteaching, private teaching, private nonteaching, and other hospitals. However, because there were so few reports from some of these categories, we dichotomized hospitals as public or nonpublic (the latter included private and other hospitals).

### Statistical Analysis

We used descriptive statistics to summarize the data and χ^2^ tests (or, for two dichotomous variables, Fisher’s exact tests) to determine associations between selected independent variables and variables reflecting the adequacy of reports. All analyses were based on SPSS for Windows version 20 (SPSS, Chicago, IL).

## RESULTS

### Patient and Laboratory Characteristics

Of the 422 pathology reports for mastectomy specimens from eligible patients, information regarding one of the BCI 2.5 data elements was not completely abstracted from five reports. These were excluded, leaving 417 reports for the final analytic sample. The median age of the study patients was 40 years (range, 22 to 100 years), with 10.8% of patients younger than age 30 years of age and 11.5% ≥ 60 years of age. The vast majority of patients (95.9%) were women. Slightly more than half of the reports (56.4%) were from public teaching hospital laboratories. Pathology residents were involved in 26.1% of the reports (Table 1).

**Table 1:**
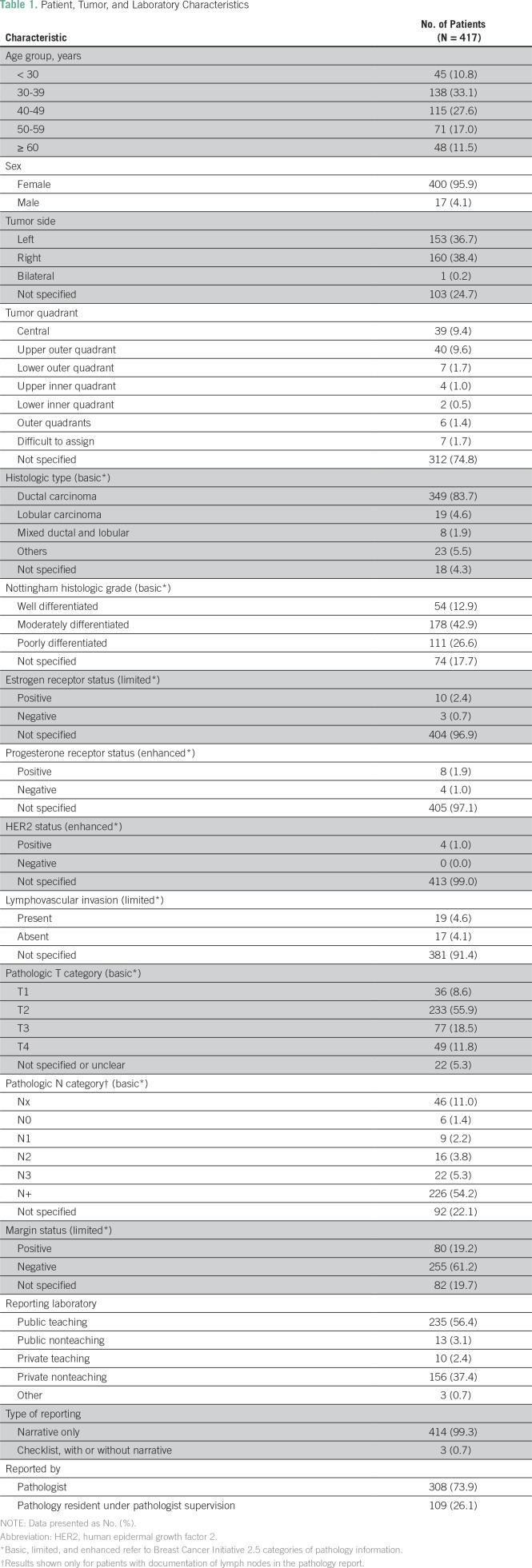
Patient, Tumor, and Laboratory Characteristics

### General Features of Pathology Reports

Only three reports (0.7%) included a synoptic summary (a template or checklist) of findings. Although all synoptic reports were from nonpublic hospitals, the association between hospital type and report format was not statistically significant (*P* = .067). Nearly half of the reports (46.0%) included relevant clinical history; this information was present significantly more often in reports from nonpublic hospitals than from public hospitals and in reports from a pathologist only than those including a pathology resident (*P* < .001). Tumor laterality and quadrant were noted in 75.3% and 25.2% of reports, respectively (Table 2).

**Table 2:**
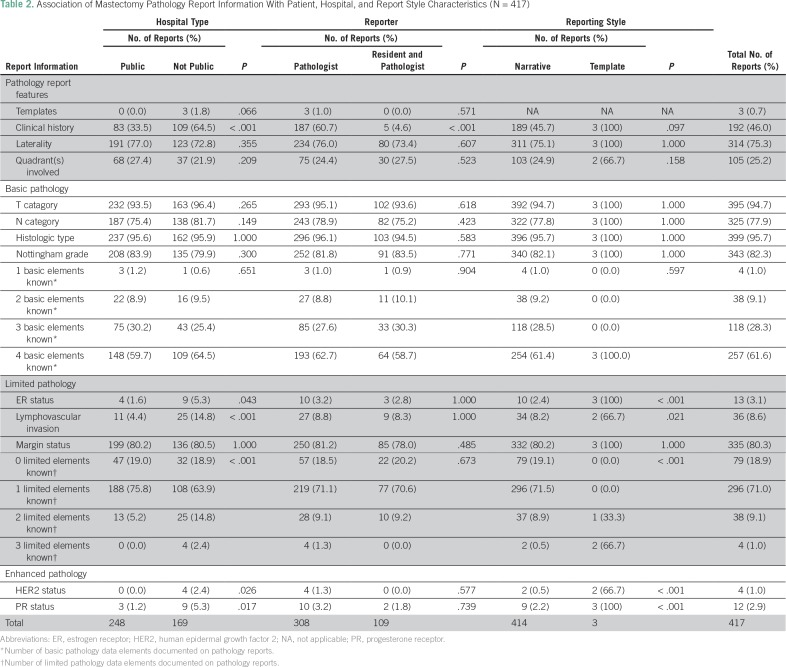
Association of Mastectomy Pathology Report Information With Patient, Hospital, and Report Style Characteristics (N = 417)

### BCI 2.5 Basic Pathology Information

The pathologic T category was noted in 94.7% of reports. The most commonly missed basic pathology element was lymph node status (present in only 77.9% of cases). Histologic type and Nottingham histologic grade were mentioned in 95.7% and 82.3% of reports, respectively. None of the independent variables (hospital type, involvement of pathology residents, or report format) were significantly associated with completeness in reporting any of the basic pathology elements. All four basic pathology elements were present in 61.6% of pathology reports, and three of the four basic pathology elements were present in 28.3% of reports. Although the number of basic pathology elements documented on the report was not significantly associated with any independent variables, it seems notable that all four evaluable elements were documented in all three reports (100%) that used a checklist but only in 61.3% of narrative reports.

### BCI 2.5 Limited Pathology Information

Overall, only 3.1% of reports mentioned ER status. This information was included significantly more often (*P* = .043) by nonpublic hospitals (5.3%) than public hospitals (1.6%) and was described significantly more often (*P* < .001) in synoptic reports (100%) than narrative reports (2.4%). Overall, the presence or absence of lymphovascular invasion was noted by 8.6% of reports, and this information was included significantly more often (*P* < .001) in reports from nonpublic hospitals than those from public hospitals (14.8% *v* 4.4%, respectively) and significantly more often (*P* = .021) in synoptic reports than in narrative reports (66.7% *v* 8.2%, respectively). Resection margin status was noted in 80.3% of reports, of these, 76.1% were negative. Inclusion of this information, however, was not significantly associated with any independent variables.

The number of limited pathology data elements (excluding those also in the basic list) included in reports was significantly higher (*P* < .001) in both nonpublic hospital reports relative to reports from public hospitals and in synoptic reports relative to narrative reports. At least two of the three limited pathology data elements were noted by 10.1% of reports overall, 17.2% of reports from nonpublic hospitals, 5.2% of reports from public hospitals, 100% of synoptic reports, and 9.4% of narrative reports.

### BCI 2.5 Enhanced Pathology Information

Inclusion of HER2 and PR status was uncommon (1.0% and 2.9%, respectively), and both were noted significantly more often in reports from nonpublic than public hospitals and more often in synoptic reports than in narrative reports.

### Other Information

Fewer than half of the reports (46.0%) included any clinical history, only 75.3% of the reports mentioned laterality of the specimen, and only 25.2% of the reports indicated the quadrant(s) in which cancer was found.

## DISCUSSION

This audit of the completeness of pathology reports of mastectomy specimens from patients registered at Ethiopia’s only oncology referral center and radiotherapy facility demonstrates gaps regarding documentation of pathology data recommended for basic-level facilities in a low-resource setting such as Ethiopia. More than one third (38.4%) of reports were found to be missing one of the four basic data elements (T category, N category, histologic type, or histologic grade) with lymph node category being absent from more cases than any other basic data element. Among patient reports with information on nodal status (n = 325), ≥ 10 lymph nodes were examined for only 53 (16.2%) of cases, suggesting inadequacy of lymph node examination for proper staging. Only 1.0% of reports included three of the data elements recommended for limited-resource settings. Several data elements were significantly more likely to be noted in reports from nonpublic hospitals than from public hospitals. Although only three of the 417 reports included checklists or templates, all three of these reports included all of the basic pathology information, and they all included at least two pathology items recommended for limited-resource settings.

Audits of breast cancer pathology reports in Nigeria from 1999 to 2008^21^ and from 2011 to 2013^22^ also described incomplete reporting of recommended pathology information. Atanda and Atanda^21^ noted that their laboratory was “at a stage comparable to that in most laboratories in Australia in 1995 before the release of specific recommendations for breast cancer reporting,” and Daramola et al^22^ recommended adoption of synoptic reporting, stating that, “The use of proformas, with the inclusion of all the main parameters, would ensure adequacy of reports.”

Observational or nonrandomized studies comparing narrative and synoptic reports from different laboratories and from different time periods strongly and consistently support the superiority of synoptic reports (especially when templates are designed optimally).^5,16,17,20,24,28-31^ For example, Appleton et al^16^ reported that completeness of tumor size and grade reporting more than doubled between 1990 and 1996, during the introduction of synoptic reporting. Austin et al^17^ compared narrative and synoptic reports of breast cancers diagnosed during 2004 and reported more complete reporting in the synoptic reports of tumor grade (86.3% *v* 100%, respectively), lymphovascular invasion (89.5% *v* 99.7%, respectively), and margin status (89.5% *v* 96.1%, respectively). The extremely uncommon use of checklists in the pathology reports we reviewed and the strikingly higher completeness of basic pathology data in these reports, despite the statistical limitations resulting from the small number of synoptic reports, support attention to this as a quality improvement intervention in Ethiopia.

Previous studies and commentaries have emphasized the multifactorial challenges and barriers to high-quality oncologic pathology practice in low-resource settings. Although checklists and templates are especially useful in reminding pathologists to observe macroscopic features and microscopic information on routine hematoxylin and eosin–stained slides and to record information they have already observed (eg, margin status; tumor size, type, and grade; lymph node status; and lymphovascular invasion), logistical, technical, and economic factors have a greater effect on limiting the use of IHC assays of ER, PR, and HER2 status. Preanalytical factors involving suboptimal fixation can reduce the accuracy and clinical value of IHC assays. The expense of reagents and equipment and a scarcity of experienced technologists are additional challenges, and the limited availability and high cost of HER2-targeted therapies undermine the practical value of enhanced pathology data in low-resource settings.^7,8,12-14^

Strengths of this study include the consistent and structured nature of this audit, the inclusion of nearly all eligible patients, and the analysis of associations between patient and laboratory characteristics and completeness of pathology reporting. One limitation is that despite having reviewed 2 years of records, statistical power is limited by the sample size and by the small number of reports with certain characteristics (especially the inclusion of only three synoptic reports). Consequently, there may be some observations that are clinically significant but not statistically significant, and some estimates of statistical significance and of prevalence for pathology report features may have limited precision. Although multivariable modeling would, ideally, help in clarifying the independence of associations of patient or laboratory characteristics with pathology report completeness, it is unlikely to provide additional insight because of the small number of reports in some categories.

Because TASHOC is Ethiopia’s only oncology referral and radiotherapy center, we are confident that our sample is representative of Ethiopians referred for oncology consultation and radiation therapy. Nonetheless, the reports we reviewed may not be generalizable to patients who are not referred to specialty care, who are treated with breast-conserving surgery, who receive no cancer treatment, or who for other reasons do not undergo mastectomy with histopathologic examination.

This audit is, to the best of our knowledge, the first systematic study of the completeness of breast cancer histopathology reporting (or for that matter, histopathology reports for any malignancy) in Ethiopia. In addition, it contributes to the extremely limited information regarding oncologic pathology in sub-Saharan Africa. Moreover, findings from this study could also serve as the baseline for evaluating the effectiveness of future programs to improve pathology reporting.

The most commonly missed basic information in pathology reports we reviewed were lymph node status and histologic grade (absent from 22.1% and 17.7% of cases, respectively). Although grade is an important prognostic factor, it is unlikely to play a significant role in postmastectomy clinical decisions for this population of predominantly node-positive patients. Nonetheless, routinely reporting histologic grade requires little time and effort, and developing experience with histologic grading will become increasingly valuable as increasing access to screening mammography and radiation therapy makes breast-conserving therapy a more realistic option in Ethiopia. Regarding limited pathology information, reporting of ER status is almost negligible, although this likely reflects lack of testing rather than lack of documentation. Developing capacity for IHC ER assays to advise decisions on hormonal therapies could be one of the most impactful laboratory priorities regarding breast cancer management.

On the basis of the findings described in this report and review of relevant literature, we recommend consideration of the following objectives: (1) organize pathology and oncology organizations to conduct ongoing audits of oncologic pathology and to guide continuing professional education programs on topics including pathology reporting, tissue handling and fixation, and relevance of pathology information to individual and population health; (2) share lessons learned and best practices regarding pathology capacity building with medical and public health organizations in neighboring countries; (3) promote use of evidence-based templates and checklists for pathology reporting in Ethiopia, which could be developed by selecting portions of the College of American Pathologists protocol corresponding to the BCI 2.3 basic and limited resource data elements; (4) promote use of electronic pathology reports to facilitate quality measurement; and (5) expand training in histotechnology (including IHC) and oncologic pathology and access to necessary equipment via collaboration with laboratories in high-resource regions, as described in published reports^32-35^ (however, such programs require sufficient resource investment to support ongoing availability of basic cancer control services).
